# Oral Health in Late Life: Challenges and Solutions

**DOI:** 10.1093/geroni/igab046.1596

**Published:** 2021-12-17

**Authors:** Xi Chen, Bei Wu, Stephen Shuman

## Abstract

Older adults face a unique challenge in maintaining their oral health due to an increased disease burden, polypharmacy, functional impairment and other reasons. The five papers in this symposium describe the oral health issues in various groups of older adults and discuss different approaches to improve oral health for older adults. Using data from the Population Study of Chinese Elderly in Chicago, the first paper examined the relationship between self-reported discrimination and oral health related quality of life and investigated how resilience mediated such a relationship among foreign-born older Chinese Americans. The second paper described the oral health concerns and related treatment needs in older adults receiving palliative care using a mixed method design. The third paper demonstrated how to use behavior change techniques to improve oral self-care skills of individuals with mild dementia and support their family caregivers. The fourth paper described a project that integrates the age-friendly health system's principles into specialty dental care to address healthy aging and oral health. This initiative helped prevent and change the false belief that aging inevitably involves deterioration in oral health. The fifth paper described the impact of COVID-19 on the management of oral health problems and access to dental care in older adults. Transformative changes in care delivery and the impact of vaccination on access to care was also explored. This symposium helps better understand the oral health needs in older adults and provides new evidence to improve oral health for these individuals.

